# Understanding humanitarian localization in Latin America—as local as possible: but how necessary?

**DOI:** 10.1186/s41018-022-00120-3

**Published:** 2022-06-01

**Authors:** Simone Lucatello, Oscar A. Gómez

**Affiliations:** 1 Instituto Mora-CONACYT, Mexico City, Mexico; 2 College of Asia Pacific Studies, Ritsumeikan Asia Pacific University, Beppu, Japan

**Keywords:** Humanitarianism, Localization, Human rights, Institutions, Disaster risk management

## Abstract

This paper questions the pertinence of the humanitarian aid localization agenda in Latin America, at least in the narrow sense embraced by the 2016 World Humanitarian Summit. Localized support has been the standard practice in the region for decades, thanks to at least two correlated factors: the Monroe Doctrine limiting intervention to the USA and regional efforts to resist such intervention. Instead, humanitarian action in the region is an example of a particular way of understating localization, mainly specialized support to specific issues, no distinction between humanitarian or development divisions, and coexistence of different response approaches, synthesizing international and local experiences that intermingle with community practices and traditions, under national government leadership. Governments, together with NGOs, civil protection, and other relevant actors from international cooperation and development, engage in crises based on a long-standing tradition of risk management at national and regional levels. Fears of abuses hidden behind the non-interference principle, human rights activism, and disaster risk management approaches to emergencies created a complex ecosystem for humanitarian localization.

## Introduction

Humanitarian crises have been central to the history of Latin America. Leaving pre-Hispanic times aside, about 90% of the continent’s original population died because of disease, violence, and exploitation during the conquest and colonization processes (Koch et al. 2019). Moreover, Latin America has been heavily affected by all kinds of disasters; there are, for instance, records of earthquakes affecting societies from as early as the fifteenth and sixteenth centuries in Mexico and Chile (Garcia Acosta 2001; Onetto Pavez 2017). The region is prone to sudden, as well as slow, onset natural hazards. According to the UN Office for the Coordination of Humanitarian Affairs (OCHA), the number of disasters and related emergencies has grown and is expected to increase in the coming decades (OCHA 2019). During the period 2000–2019, 152 million people were affected by 1205 disasters in the region. Floods, hurricanes, earthquakes, climate change, unplanned urbanization, and accelerated population growth are significant risks and vulnerabilities in the region, including the small island developing states in the Caribbean. Even though Latin America has not suffered any great or calamitous famines since the end of the nineteenth century (de Waal 2018), drought still affects mainly rural populations. Outbreaks of infectious diseases like dengue, chikungunya, cholera, and recently Zika virus and the COVID-19 pandemic are other disasters that have affected Latin-American populations. Armed conflict and other forms of violence have been a constant challenge, accompanied by different forms of forced displacement. We offer in this introduction an overview of the main regional spots for humanitarian attention and discuss the perspectives for localization and humanitarian action. Given the large number of countries in the region, only few of them will be analyzed in detail in the article: the Northern Triangle (Central America), Mexico, Venezuela, and Haiti. Haiti is the only Caribbean island, given its very particular condition, that is mentioned in the article.^1^

The region is usually at the periphery of the humanitarian system’s attention. Historical accounts of humanitarianism, such as Barnett (Barnett 2011) and Davey (Davey 2013), merely mention the region in passing. More recently, since the Inter-Agency Standing Committee and the European Commission started publishing in 2014 the INFORM reports on “risk assessment for humanitarian crises and disasters,” Latin-American countries have never appeared among the twelve countries with the highest risk. Mexico appears among those with the highest values in the hazard and exposure category, while Haiti is among the most vulnerable (INFORM 2020). In the Global Humanitarian Assistance reports produced by the Development Initiatives since the year 2000, Colombia, Guatemala, Haiti, and Honduras have occasionally appeared among the more affected countries, and only Haiti after the 2010 earthquake as one of the top recipients of humanitarian assistance—Venezuela, has become a source of concern lately.^2^

About Haiti, it is worth considering that the country may be an exception to the overall Latin-American way of dealing with humanitarian responses. The amount of international attention, both in the form of international cooperation, military deployment, UN agencies myriad of missions and interventions, and other regional factors like the involvement of Brazil and other Latin-American actors after the 2010 earthquake in humanitarian responses to the island, gives to Haiti a “unique” status for the humanitarian situation. In the midst of multiple crises, from peace and security challenges to a humanitarian emergency, Haiti lies in the center stage of a paradigmatic case for the region, where humanitarian “consistency” is fragmented or not sustained over time (UN-OCHA, 2022).

For Central America, conflicts in the 1980s resulting in tens of thousands of deaths and a refugee crisis are included among the Cold War dynamics and peace studies, but not necessarily in the annals of humanitarianism.^3^ In the 1990s, Honduras and Nicaragua appeared as major recipients of assistance after 1998 Hurricane Mitch, although then the pledges were mostly not humanitarian — see, for instance, Gómez (Gómez 2018). Development Initiatives (2016) reports Colombia, Ecuador, and Venezuela as part of the list of *forgotten* crises identified by the European Commission. Lately, El Salvador, Guatemala, Honduras, and Mexico have also been mentioned concerning violence-related problems.

In sum, the region is affected by different kinds of disasters whose manifestation is expressed in many forms. Exposure, vulnerabilities, and cascading risks do represent severe challenges for humanitarian responses and localization; we argue that underlying this duality of fragility without significant international attention is the product of long-standing localized institutions and practices of (humanitarian) crisis management. In the following section, we explain why the Grand Bargains’ (GB) aid localization proposal is too narrow when seen from Latin America; instead, we propose that the region’s experience shows how the concept of localization in the Gran Bargain can be expanded by encompassing the comparative advantages that exist throughout the regional humanitarian ecosystem—playing out the various strengths and utilizing them to highlight the characteristics of the region. For this, we explain how historically the Latin-American system could evolve in a localized way, mainly because of the Monroe Doctrine (MD) and the rest of the region’s push to resist it. Then, we characterize the resulting humanitarian institutions, shaped by a mixture of a new rhetoric linked to violence and forced displacement (both urban and rural violence), disaster risk management, climate change, and the war on drugs and renewed religious beliefs contributing in different ways to a unique practice in all kinds of crises. Governments’ ability to deal effectively with disasters is uneven, although they recognize their responsibility to meet the challenges of assisting and protecting victims. Over time, each country has defined systems for national disaster management and criteria for engaging the international community in humanitarian action and institutional capacities to prepare for and deal with disasters. Moreover, the region has been experiencing a forward momentum through which inequalities, continuous political instability, violence, and an unprecedented series of disasters undermine traditional public security concepts. All these events have shaped new forms of addressing the humanitarian “localization” experiences in the region both at the political and technical level.

Based on official documents and empirical research in different regional summits and mechanisms for humanitarian action, we argue that humanitarian localization in Latin America rests upon a mixture of ideological, political, and technical experiences, influenced by different interventions streams. It also depends on how states, local governments, civil society organizations, and communities understand and work the humanitarian concept and practice. Even though this may not be so new, it is relevant to point out that the variety of experiences used by regional governments directly influence many Latin-American countries’ political engagement in new forms of cooperation within the region itself. Recent developments and impacts of the COVID crisis in the region also underlined that universal health-care systems in the majority of Latin-American countries have significant gaps. COVID-19 began as a health emergency but has since evolved into a humanitarian disaster. A political declaration on a regional level was signed in the region at the end of 2021 with the support of international organizations such as the WHO, the EU, and other international traditional donors of humanitarian aid in the region^4^. Prompt rehabilitation, long-term, inclusive, and resilient COVID-19 actions acknowledge not only inequalities exist in the region but also social investment; health protection is required to be fully implemented in the region (The Lancet 2021).

### The narrow conception of humanitarian aid localization^5^

The localization of humanitarian aid has received significant attention since the 2016 World Humanitarian Summit (WHS). Aid localization is about giving more support to national first respondents, making humanitarian aid “as local as possible, as international as necessary,” echoing concerns about the amount of international humanitarian assistance directly reaching local nongovernmental organizations (NGOs). Aid localization has made it to the yearly UN-Secretary General (2017; 2018; 2019) reports on “Strengthening of the coordination of emergency humanitarian assistance.” Annual independent evaluations of the Grand Bargain include aid localization among the commitments in which progress is visible (Derzsi-Horvath et al. 2017; Metcalfe-Hough et al. 2018; Metcalfe-Hough et al. 2019). Indeed, Metcalfe-Hough, Fenton, and Poole (Metcalfe-Hough et al. 2019, 31) report that there is “a growing *normative* shift towards” aid localization, suggesting signs of systemic improvement, although much remains to be done.

Positive as the picture seems to be, the Grand Bargain version of aid localization looks too narrow for what localized action implies in dealing with major and minor crises. Two main issues are the local organizations it focuses on and the breadth of resources it considers. About the former, many of the Grand Bargain proposals concentrate on Global South NGOs. For instance, the “Charter for Change,” mentioned in the WHS outcome and later by the UN Secretary-General (2017), is devised explicitly for international actors and the way they work with southern-based NGOs, who are the charter main signatories. Other authors suggest a broader understanding of the local, but the emphasis on the nongovernmental sector is prominent (Barbelet 2018; Schenkenberg 2016; IFRC 2015; Wall and Hedlund 2016). Emphasis on NGOs is understandable under the current international humanitarian order, but it is less so when the local response to emergencies is the focus of the attention. After all, ordinary people believe that the state has the responsibility of their protection and expects their governments to fulfill this responsibility. Therefore, you would imagine that localization initiatives should transfer initially, and as much as possible, capacities from the international system to states, not to NGOs.

Reasons of principle underlie the emphasis on NGOs instead of the government. Traditional humanitarianism expects governments not to be always willing to support embattled populations (IFRC 2015, 21; Barbelet 2018; Schenkenberg 2016). No matter how capable states are, negligence or power abuses cannot be discarded, particularly when governments’ political and social standing is at risk because of the crisis. Schenkenberg (Schenkenberg 2016) frames this as the “sovereignty agenda,” highlighting how in armed conflict settings, curtailment of access, and other fundamental freedoms could be expected, as sovereignty clashes with humanitarian principles—i.e., independence, neutrality, impartiality, and humanity. First of all, when “local” refers to governments, principles of independence and neutrality are automatically discarded since they precisely address the relation between aid and parties of conflict—for other types of crises, these two principles are not as relevant. Humanity and impartiality apply more broadly to all kinds of emergencies, and critics suggest that local response could be biased for ethnic, geographical, or religious reasons, among others, leaving some populations vulnerable. Schenkenberg (Schenkenberg 2016, 23) summarizes the challenges in “intentional (e.g., from a conscious decision to privilege a particular group), unconscious (such as a repetition of culturally normalized patterns of exclusion), or driven by a (perceived) fear of immediate or future retaliation by local power actors towards the organisation, its members and/or their families.” Sovereignty concerns are thus expected to come along with localization efforts, which is the Latin-American experience.

Besides, coming from the High-Level Panel on Humanitarian Financing (2016), aid localization emphasizes the issue of resources. In 2014, only 0.2% of the international humanitarian assistance directly reached local NGOs, but, while grim, the figure is only meaningful as far as you remain on the narrow humanitarian sphere of action. For example, Development Initiatives (2018, 30) reports how in the 20 countries receiving the most international humanitarian assistance, this assistance accounts for only 4.6% of the international resources, which is 1.7% of the full resource mix available. Domestic government non-grant revenue accounts for 63%; international financial flows different from official humanitarian assistance, for instance, remittances, are much larger. Remittances usually outweigh official development assistance for the region but also can be highly unstable depending on the economic conditions of sending countries. If this is the case for the most prominent humanitarian aid recipients, in wealthier regions such as Latin America, international resources’ insignificance is more accentuated. According to the World Bank database (W 2020), the net official development assistance (ODA) received by the region as a share of the gross national income has decreased from 0.75% in the 1960s to below 0.2% in the last decade—even after excluding high-income countries (Development Initiatives 2018). Remittances are more critical than humanitarian assistance, reaching affected people directly and empowering them (Wall and Hedlund 2016). In a country like El Salvador, ODA represents 1% of the GDP, which pales against 21.4% coming from remittances (BBVA 2019). The financial approach to localization of crisis management is utterly myopic to the reality of financial flows in Latin America.

Localization needs a new understanding to be of any practical use, at least in the region. We propose that the history and experience of Latin America can offer some insights into the way crisis management has remained local despite intervention pressures while highlighting some of the challenges in realizing the common goal of protecting humans despite borders and nationalities.

### Historical background of localized humanitarian action in Latin America

The history of localized humanitarian action does necessarily diverge from usual humanitarian narratives. Traditional narratives are asymmetrical, as humanitarian action usually flows from great powers towards the south, where crises occur. Agency is almost exclusively attributed to the intervening organizations, while passivity and lack of capability are assumed about the receiving side, which is still the basis for humanitarian action nowadays. Local actors cannot be expected to share this view, and thus, the key to tracing back the root of localization is identifying resistance and contestation to portrayals of fragility and incompetency. The main difficulty in tracing this contestation is that actors from the Global South would not do it inside a “humanitarian” framing but resort to other frames. The lack of attention to Latin America across the humanitarian literature indicates that is the case. Identifying contestation framings is thus the first task to understanding localization.

Colonialism and imperialism have been the primary sources of fragility and incompetency stereotypes about the South. The fragility and incapacity of locals to protect themselves from all sorts of threats were critical to justify the colonial regime’s existence. If locals could not address humanitarian concerns, the imperial power would provide the required protection as part of its civilizing mission (Paulmann 2016). However, different from regions more traditionally identified with humanitarian action today, most Latin America was already free from the colonial rule when humanitarian practices and institutions started to coalesce during the nineteenth century.^6^ The region resisted European attempts to recolonize and maintain control over newly independent states, both through force and other means (Friedman and Long 2015). Resistance included two essential ingredients: the US Monroe Doctrine (Sexton 2011) and Latin-American states’ efforts to nurture their capacities, showing to the world how civilized they already were (Obregón 2006). Similar observations related to peace missions can be found in Hirst (Hirst 2017). Localizing humanitarian action in the region was thus closely linked to maintaining sovereignty and international intervention fears.

The Monroe Doctrine, and its subsequent corollaries, which in 1823 asserted that the American continent was not anymore subject to European colonization, was critical to keep at bay European pressures. At the same time, it already included the seeds of US imperial ambitions, as the doctrine was applied “as an expansionist principle over the Americas” (Scarfi 2014). This anti-colonial imperialism shows the ambiguous standing of Latin America vis-à-vis the USA (Williams 2009). While the doctrine offered an umbrella of protection, it maintained the civilizational, moral justification for intervention (Ninkovich 1986). From those days onwards, lofty claims of protection, particularly against abusive regimes, would continue to disguise US interventionist character in the region (Falk 1996).

Paradoxically, US imperial anticolonialism inhibited the spread of humanitarianism in the region. On the one hand, being the regional hegemon, the USA could not pose as Latin America’s savior because interventionism backfired and nurtured a culture of anti-Americanism widely shared by the public. It is telling how book-length accounts of American humanitarianism such as Poter (Poter 2017) and Irwin (Irwin 2013) barely mention Latin America. Observe that Poter (Poter 2017) includes a chapter on Cuban refugees, but this is not part of any civilizing mission. Instead, the Cold War changed the humanitarian action logic, as crises affecting Communist regimes served as propaganda against the enemy.^7^ While the Soviet influence posed a challenge to the Monroe Doctrine, the use of humanitarian crises to show Communism moral bankruptcy did not work for the rest of the continent, as the USA supported the regimes generating violence, repression, and forced displacement in the region.

On the other hand, Latin-American governments could concentrate their efforts against foreign intervention on the USA alone, thanks to the doctrine. Early evidence of the complex relationship between humanitarian action and self-determination can be seen in the earthquake that rattled Venezuela in 1812. Then, the country was struggling to get its 1810 independence declaration recognized by the world. Venezuelan leaders expected the USA to “lift the embargo so that private citizens could participate in a human effort to ship goods and relief to Venezuelan ports. Instead the US Congress voted to send a mere fifty thousand pesos worth of earthquake relief” (Ewell 1996, 20). The nascent state leadership understood that they should not rely on foreign aid but develop their own resources.^8^

Latin-American agency to resist and contest US intervention included transforming the interpretation of the Monroe Doctrine. Scarfi (Scarfi 2014) argues that South American politicians and intellectuals played an essential role in putting forward versions of the doctrine between 1898 and 1933, which helped to shift its meaning “1) from a principle of intervention to one of non-intervention; 2) from a unilateral to a multilateral doctrine; 3) from a political to an international law principle; 4) from a national to a hemispheric principle.” The first shift reflects the contestation that we have discussed, while the other three evidence early signs of the shape liberal humanitarianism would take after the end of the Cold War (Barnett 2011). This mix of resisting intervention but recognizing multilateralism’s role is fundamental to understanding why the region has not been perceived as abusing the non-intervention principle (Coe 2015). The primary means for this transformation were informal alignments, international organizations, and early engagement in international law’s evolution. Finally, in the 1930s, the USA committed to stopping intervention in the Americas in the context of regional conferences (Scarfi 2014), although, as we described above, US interventionism continued during the Cold War and beyond.

International organizations and international law’s vital role also help explain how humanitarianism failed to take root in the region. First, most Latin-American countries very early created national societies of the Red Cross. Established in 1863, the Red Cross is the oldest humanitarian organization; Latin-American countries were fast to join this movement, with societies created in Peru and Bolivia (1879), Argentina (1880), El Salvador, and Costa Rica (1885). The nature of these organizations was initially about the provision of medical relief during armed conflicts but quickly moved into other areas of health and relief provision. At least equally important, their existence served as an appeal for recognition by the international society of their sovereignty and civility, in as much as they implied a commitment to follow the rules of war—although hardly was the region accepted on an equal standing by the international society (Schulz 2014). Early creation of Red Cross societies does not necessarily imply a unique approach to humanitarian action but an early start in its localization.^9^

Second, the long tradition of contributions through international law empowered a different approach to deal with suffering and atrocities in Latin America: human rights. Not only did the region plays an essential role in the framing of the Universal Declaration of 1948 (Glendon 2003), but it was also crucial in actually starting the practice of human rights in the 1970s (Moyn 2010; Kelly 2018). The background of human rights emergence in the region was the multiple US-backed authoritarian regimes, one reason why human rights were dropped out from Inter-American debates during the first part of the Cold War (Sikkink 1997). When NGOs from Britain, Canada, and Ireland tried to help in El Salvador during its civil war in the 1980s, they found already two major local approaches through which the Anglo-Saxon idea of humanitarianism had to be adapted, one of them being human rights (Desgrandchamps et al. 2020). International support did exist, and nonintervention did not mean silence to state atrocities. Yet, the region’s activism was the one doing the job and taking the credit for overturning regimes and pressuring for a change in the US posture.

International law was not the only way through which the region resisted intervention and humanitarian framings. The second framework that O’Sullivan found in Latin America was the Liberation Theology, which emanated from the Catholic Church as a plea to confront social inequities and to support active resistance against structural injustices. As an international network, the church played a pivotal role in channelizing concern and solidarity towards those suffering from acute and chronic suffering in the region. Liberation Theology is an extreme example of this power to mobilize society in the face of disaster. It is symptomatic of the church’s crucial role in the nation- and state-building processes in the region, ensuring a minimum of respect to the lives, livelihoods, and dignity of the vulnerable. Yet, this was beyond what can be called humanitarian action, and the local churches would not call it that. A more in-depth analysis of such historical posture of the local church is beyond this paper’s reach. Still, we suggest that the church’s support in times of suffering and upfront resistance, as with the Liberation Theology, contributed to creating institutions dealing with problems that could become humanitarian crises. Such institutions are the focus of the rest of the paper.

### The localized “humanitarian” system in Latin America

An approximation to the localized humanitarian ecosystem in Latin America points to coexisting humanitarianism models, contributing to specific and ad hoc patterns of humanitarian action and responses, which are reproduced on a different scale across the region with different results (Lucatello 2017). Civil war and dictatorships between the 1970s and 1990s left traces of institutional instability in many countries in the region. Latin America also experienced a transition to democratic institutionalization over the past 30 years and substantial economic neoliberal reforms (Washington Consensus). This experience created an interesting setting that prompted updates of the institutional frameworks developed for emergency response to all kinds of disasters, including violence and displacement due to narcotraffic-related violence in Colombia and Mexico. Thus, the region’s current humanitarian system combines sociopolitical, natural, and economic determinants through its emergency-specific institutions.

The ecosystem is also a by-product of the intense academic thinking that the region produced in the past decades and that has contributed strongly to the understanding of risk concepts and vulnerability.^10^ For example, la RED, involving different stakeholders, has played a leading role not only in Latin America but also throughout the rest of the world, in promoting understanding of disasters and disaster risk as a social construct. The trans-disciplinarity of the “la RED” thought had important impacts on the development of the integrated research on disaster risk approach. Mainly, the concept of risk was transformed from a plain physical deterministic notion into a paradigm in which recognition of the social construction of risk, which is conditioned by societal decisions, demands, needs, perceptions, priorities, and practices, is helping to unveil the need to redefine disaster risk management (DRM) (Alcántara-Ayala 2019). The legacy of this movement and thinking shaped the way many governments are currently dealing with risk conceptualization in Latin America. The debate promoted by “la RED” stimulated and introduced a stronger social studies approach to the risk and to define new forms of intervention and management in the sphere of disaster risk prevention and mitigation. These groundbreaking concepts can be summarized into five major notions: (1) disasters are not natural but socially constructed; (2) the inherent nexus between disaster risk, development, and the environment; (3) the significance of small- and medium-sized disasters and extensive and intensive risks; (4) disaster risk management at the local level (Lavell et al. 2013); and (5) integrated disaster risk research and the need for forensic investigations of disasters (Alcántara-Ayala 2019).

**Fig. 1:**
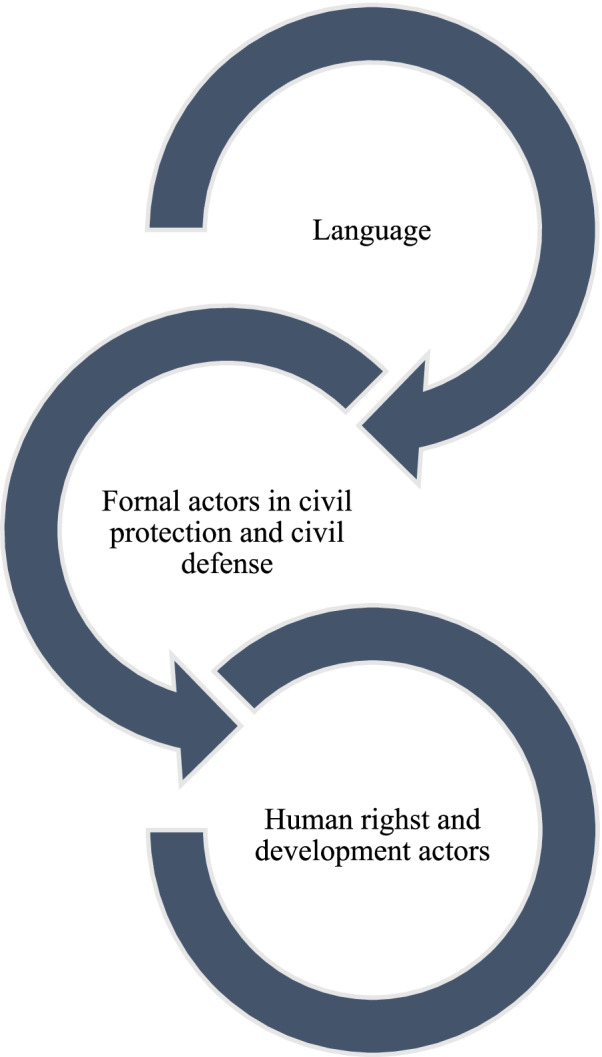
Shaping factors in determining humanitarian localization. Source: authors’ elaboration

Diverse institutional efforts have promoted regional cooperation mechanisms, producing different results at local and national levels (Churruca 2015). That said, the emerging institutions do not form a consolidated “humanitarian system.” They belong to different parts of the state, emphasizing national disaster management systems, drawing heavily from civil protection (Bragg 2014). Indeed, Latin America is considered a forerunner in disaster risk management (DRM), benefiting from the modernization of legal frameworks and organizational structures. The region is proud of its risk management models, characterized by robust national and regional coordination systems (MIAH 2015). Table 1 presents a list of national institutions and the year of creation. In general, there are currently 23 national civil protection systems, more than a dozen disaster risk prevention and management institutions, such as the Coordination Centre for Natural Disaster Prevention in Central America (CEPREDENAC), Mexico’s National Centre for Disaster Prevention (CENAPRED), the Caribbean Disaster Emergency Management Agency (CDEMA), Guatemala’s National Coordination for Disaster Reduction (CONRED), and the Permanent Commission (COPECO) of the Inter-American Network for Disaster Mitigation (RIMD). Over the past four decades, countries in the region have shifted from civil defense to civil protection schemes to attend emergencies. This was further influenced by the United Nations Decade for Natural Disaster Reduction which proposed an integrated global approach to mitigating the effects of disasters particularly in developing countries. Therefore, mapping institutions in the region that attend humanitarian issues, we can say that the region shows a complex dynamic and layers of systems, including the military, the Fig. 1 International and local Red Cross, and more than 200 NGOs working in the field (Lucatello 2017) (see Annex 1).

**Table 1:** Institutions for DRR in Latin-American countries (selection)

Country	National authority and legislation creation date	Regional mechanisms for DRR
**South America**		
Argentina	National System for Integrated Risk Management and Civil Protection (2004)	UNASUR (2011)
Bolivia	National Program for Risk Management (2000)	Andean Strategy for Disaster Risk Management — EAGRD/CAPRADE (2002)
Brasil	National System for Civil Defense and National Centre for Monitoring and Early Warning of Natural Disasters (CENAD) 1993 National Plan for Risk Management and Disaster Preparedness (2004)	UNASUR
Chile	Oficina Nacional de Emergencia del Ministerio del Interior y Seguridad Pública (ONEMI) 1983	UNASUR
Colombia	National Program for Disaster Risk Management (SNGRD) (1982)	UNASUR Andean Strategy for Disaster Risk Management—EAGRD
Ecuador	National Ministry for Risk Management (1991)	Andean Strategy for Disaster Risk Management—EAGRD
Paraguay	Ministry of National Emergencies (SEN) (2005)	UNASUR
Perú	National System for Disaster Management (SINAGERD) (1990) National Plan for Disaster Risk Management PLANAGERD (2014–2021) National System for Civil Defense (SINADECI), 1972	UNASUR Andean Strategy for Disaster Risk Management—EAGRD
Uruguay	National System for Emergencies (SINAE) (2009)	UNASUR/PROSUR
Venezuela	National Administration for Civil Protection and Disasters (1999)	UNASUR
**Central America**		
Costa Rica	National Commission for Risk Prevention and Emergency Responses (CNE) (1969)	CEPREDENAC (1987)
El Salvador	National Administration for Civil Protection (1993)	CEPREDENAC
Guatemala	National Commission for Disaster Risk Reduction (CONRED) (1996)	CEPREDENAC
Honduras	Permanent Commission for National Contingencies (COPECO) (1974)	CEPREDENAC
Nicaragua	National System for Prevention, Mitigation, and Disaster Response (SINAPRED) (2000)	CEPREDENAC
Panamá	National System for Civil Protection (SINAPROC) (1982)	CEPREDENAC
**North America**		
México	National System for Civil Protection—SINAPROC (1986)	
**Caribbean**		
Cuba	National System for Civil Defense (1987)	
Haiti	Direction de la Proteccion Civil (1999)	CEDMA (2005)
Jamaica	Natural Hazard Risk Reduction Policy (2005), the Building Code Bill (2013), and the Disaster Risk Management Act (2015)	CEDMA (2005)
Puerto Rico	State Agency for Emergency and Disaster Management (1979)	CEDMA
Dominican Republic	National System for Emergency and Security (2011)	CEPREDENAC-CEMDA

Source: Own elaboration

These institutions, created initially to deal only with disasters, have become necessary support in all types of crisis situations because of their capabilities. For instance, during the 2020 COVID-19 pandemic, Health Ministries teamed with civil protection and other governmental agencies to monitor the disaster and provide relief. They offer a platform to coordinate different government branches and surge capacity when the situation requires it. They are the backbone articulating a broad range of local actors active in crisis response. Moreover, there is also a layer of regional organizations that support embattled countries and smooth coordination. For example, since 1987, the CEPREDENAC has been active in Central America, following the mandate established by the Secretariat of the Central American Integration System (SICA). Furthermore, the Caribbean Community (CARICOM) has benefitted from the work of the CDEMA. Countries in South America have recently established the High-powered Committee for Integral Disaster Risk Management of the Union of South American Nations (UNASUR). Even though UNASUR is currently undergoing a slow disintegration process with just 5 out of 12 original members, it keeps having a relative influence on disaster risk management within South America through its technical committee and its recommendations to member states. UNASUR will be probably be taken over by a new organization called the Forum for the progress of South America, also known as Prosur, that has a mission to promote cooperation and development in the subcontinent (PROSUR 2020).

The region’s experiences in managing disasters thus show that it has its own modus operandi.^11^ The interrelation between government institutions, other local actors, and international organizations is dynamic because humanitarian contexts are unique and ever evolving. Joint work depends on the specific characteristics of each crisis, mediated by particular features of local institutions. Several of these features can be identified. The first one is the discourse or language, i.e., how concepts and terms are used locally to make sense of humanitarian response. Localized action is accompanied by terms and concepts that shape decisions and actions aimed at supporting humanitarian action (GALA 2018). Humanitarian organizations working in the region, both international and national, have developed overtime contextual and cultural notions of “local humanitarian needs,” shaping partnership and forge assistance strategies. Yet, these are not always presented as humanitarian, but as human rights violations, for instance. On the other hand, local offices in the region offer civil protection or disaster risk management, under a humanitarian aid perspective, where it is conceived as a distribution of needs to alleviate suffering when a disaster strikes. This is rather a mechanical response but not a comprehensive response where development factors, inequalities, and other key humanitarian political, economic, and social determinants are considered for attending the emergency. (e.g., the nexus between development-peace-humanitarian response). Those factors are left for national and local governments to address through the recovery.

While global agreements such as the Sendai Framework for DRR and regional mechanisms like the MIAH^12^ influence the discourse, long-standing local agency in emergencies has been fundamental in drafting such global frameworks (Alcántara-Ayala 2019), so they are not seen as impositions. On the other hand, any mention of “humanitarian intervention” is resented by Latin-American governments since the term remits to US-backed military occupation and the European colonialism discussed in the previous section.

A second feature is civil protection and civil defense organizations’ central role in responding to different threats and emergencies. Civil protection actors contribute to risk management’s broad agenda, including humanitarian action as part of their traditional focus on disaster preparedness and response.^13^ They also stimulate governments’ engagement in improving emergency policies, such as the follow-up to commitments included in regional and national platforms for DRR. Sometimes they also participate as coordinators in major emergencies, thereby facilitating the actions of different actors involved. We can argue that a Civil Protection’s customary focus on preparedness and response and its involvement in the broader DRR agenda are important features for understanding humanitarian localization in the region. These institutions, while not unique to the region, have helped formalizing the role of different branches of the government in covering the whole crises management cycle.

The third feature of Latin America’s humanitarian institutions is the supportive role of human rights and development organizations. The literature available on this topic is vast and beyond the scope of this paper; however, it is worth describing in very general terms some of the multiple ways in which those organizations become part of the humanitarian ecosystem. On the one hand, development organizations are often dependent on the local needs to provide support in coordination with local governments, offering a safety net to humanitarian aid beneficiaries. On the other hand, they also help uphold humanitarian law and other international standards and support the implementation of transition agreements following political instability (Studer and Fox 2008). They also operate within a political and policy framework elaborated in close cooperation with the national authorities and UN bodies to ensure human rights protection.

Stakeholders’ perceptions of how other humanitarian actors deliver services in terms of time, motivation, and client orientation play a role in shaping local humanitarianism. For example, international humanitarian organizations can be perceived locally as *parachuting in*, with little knowledge of the local context and a strong focus on a pre-determined agenda, driven by different humanitarian imperatives (GAUC 2019). The so-called “first in-first out” deployments of international staff on short-term contracts and implementing programs with short-term funding cycles also affect how affected communities understand international humanitarian action.

It is worth saying that defining all these local actors as “humanitarian” can be misleading. The scope of action of the multiple actors involved in humanitarian crises usually goes beyond emergency relief. This is often the case of organizations like religious groups, the health sector, private foundations, or human rights advocacy groups, as well as development organizations that primarily identify themselves not as “humanitarian” but rather momentarily as first responding institutions to disaster relief. Engagement in the relief allows them to later act in line with their overall mission, principles, or long-term objectives, which are not necessarily humanitarian. A clear example is the recent economic and social crisis in Venezuela, leading to the exodus of more than 3.4 million persons. Most have left in the past 12 months and have gone to Colombia, Peru, Brazil, Ecuador, and Chile, the main receiving countries. The magnitude of the migration makes it among the biggest displacement globally. During this crisis, multiple local organizations have intervened in the provision of humanitarian assistance, applying standards and principles of humanitarian action but not clearly coordinated, and definitively not self-identified as humanitarian.

There have been efforts to generate a humanitarian identity in the region, with modest results. The leading promoter has been OCHA, which has a relative presence in Latin America compared to other regions. OCHA focuses on supporting existing platforms, brokering and facilitating the international humanitarian system’s work, mostly concerning disaster risk management and responses. A relative exception is the work of the International Humanitarian Assistance Mechanism (MIAH). While under the “humanitarian” frame, MIAH regional meetings from 2008 to 2019 (8 high-level regional meetings) brought about acknowledgments from governments regarding the need to look for an integral vision of DRR, with a special focus on vulnerability reduction, capacity creation, improved information access, institutional strength, and better responses to the challenges of preparedness and actual emergencies (MIAH 2017). Such emphasis is particularly important, as such integral version of the DRR management cycle has traditionally resisted approaches that mainly address relief, a reason not to see themselves as humanitarian in the vernacular.

Occasionally, the MIAH meetings have also helped evidencing regional resistance against international humanitarianism. This was the case during the consultations for the 2016 World Humanitarian Summit, which built upon MIAH’s work. A Regional Steering Group (RSG) co-chaired by the Guatemalan Government and the United Nations Office for the Coordination of Humanitarian Affairs Regional Office for Latin America, and the Caribbean (OCHA-ROLAC) guided and organized the regional consultation process, which concluded in May 2015. The members of this steering group include member states, previous hosts of the MIAH process, subregional intergovernmental agencies, United Nations agencies, the International Federation of the Red Cross and Red Crescent Societies, members of the academic sector, and the private sector and civil society organizations. As a result of these consultations, the region emphasized its traditional agenda of identifying, assessing, and monitoring disaster risks, enhancing early warning, and strengthening disaster preparedness for effective response at all levels. However, it was impossible to recognize some outstanding concerns about violence or internal displacement due to narcotraffic and other problems related to violence. Again, national actors who are facing severe humanitarian crisis like Mexico or Central American countries, do not consider these issues as part of the broader humanitarian agenda. Instead, governments consider these problems belonging to their security agendas, as we explore in the next section.

### The limits of a localized “humanitarian” system

In Latin America, the ecosystem of “humanitarian” local organizations goes beyond the traditional humanitarian-development divide applied to local actors. Engaging with multiple issues underlying crises, such as response, resilience, and governance, the region has developed a modus operandi in its own terms, consistent with the already described shaping features. This order works relatively well in disaster situations, but the performance in displacement and violence situations evidence some of the limitations of local institutions.

Two contemporary crises illustrate the challenges of addressing displacement in the region: the *caravans* from Central America, crossing Mexico towards the USA, and the Venezuelan migrants. As a reference, the cases illustrated could be also work as an example of the Triple Nexus (Humanitarian Development Peace) that envisions more collaboration and coordination among actors in the sectors of development cooperation, humanitarian action, and peacebuilding.

The former is the current humanitarian corridor provided by Mexico along the Guatemala border. As a country of transit, Mexico had tolerated these populations’ movement yet, pushed by the Trump administration, and continued under the current Biden administration, it has been forced to confront the challenges of becoming the migrants’ final host. The Mexican government, fearing the loss of trade opportunities with the USA, agreed to “temporarily” house thousands of migrants in extremely unsanitary and unsafe conditions, while the US systematically avoided its legal obligations under US federal law to process asylum applications (Jawetz 2019). At the same time, Mexico implemented a development program with Central American countries of the Northern Triangle—i.e., Honduras, Guatemala, and El Salvador—in an unprecedented aid economic package of roughly one billion US dollars. On this particular occasion, facing Trump’s threats to impose tariffs that could devastate its economy, Mexico agreed to let migrants stay on its soil as the USA processes their asylum claims and to promote international economic cooperation with Central American countries. Within the security-development nexus, Mexico has also deployed its National Guard to deter migrants’ caravans from the Northern Triangle to cross the border.

In the caravans’ case, Mexico had to react to a US threat, but it does not have the infrastructure, or the resources, to house hundreds of thousands of refugees and asylum seekers. The country’s improvised response follows a short-term policy on migration and humanitarian needs for refugees. The current crisis has also evidenced the limits of emergency improvisation, which was based more on external pressures (mostly exerted by the Trump and Biden administrations) rather than a coordinated and integrated response to a humanitarian crisis. The Mexican government could not deal with the situation alone, and so, we need to understand better how informal and nontraditional actors are engaged in building responses with traditional actors (Leuter 2020).

The caravans’ crisis case also showed how a bureaucratic approach to humanitarian response is very narrow in the scope. The coordination challenge was unexpected, overwhelming, and complex. In recognition of this, humanitarian leadership could have been strengthened further at the outset of the operation. A more focused and better-defined coordination capacity among regional humanitarian actors was needed to ensure cohesion between the response operation’s strategic and operational levels. The overwhelming military assets deployed by the Mexican government, only aiming for relief provision, failed to include socio-economic and environmental dimensions of the crisis and engage the local humanitarian community and other stakeholders.

Another interesting lesson for localization in the Mexican case with the Northern Triangle crisis is how various partnerships can evolve during surges and prolonged responses, continuing long after the acute crisis phase has ended. Despite the serious humanitarian impact of organized armed violence in the area, different actors have been working and implemented schemes of cooperation, like the Development Plan for Central America, which came into force in 2019. Promoted by Mexico, the new plan is rather ambitious, including goals related to “citizen security,” “social cohesion,” and “peaceful co-existence.” The designed implementation of the program incorporates a new integrated approach the sustainable development. Moreover, the Comprehensive Development Plan for El Salvador, Guatemala, Honduras, and south and southeast Mexico also includes the promotion of universal access to social rights, fostering resilience to climate change, and guaranteeing rights throughout the entire migratory cycle (ECLAC 2019). The shift towards a new integrated approached to humanitarian relief must be evaluated in the light of many stressors, including recent disasters associated with tropical cyclones in 2020. However, it represents a new framework for action that include humanitarian components and the nexus between development (mostly the Agenda 2030) and assistance.

Some similarities can be found in the Venezuelan humanitarian crisis, which has seen millions of people fleeing the country and moving to Colombia, Perú, Brazil, Ecuador, and Chile. These countries have invested themselves deeply in supporting displaced populations, partly inspired by a long history in which they have been on the sending side (Gómez 2019). They have come up with “humanitarian infrastructures” to confront the challenge, although these efforts are ambiguous in the use of migrant and refugee frameworks as part of their management strategies for control and freedom (Moulin Aguiar and Magalhães 2020). On the other hand, the USA imposed sweeping sanctions on Venezuela, including a freeze on all Venezuelan government assets in the USA and a bar on transactions with his government. Trump’s government attempted to organize a humanitarian intervention that was promptly dismantled by the Venezuelan government and disapproved by many states of the region, like Mexico. Observe how regional actors have been absent from these responses, losing the opportunity to expand its catalytic role for collaboration. The state remains the central humanitarian actor.

The centrality of the government is even more absolute in addressing violence, hindering traditional humanitarian approaches. The Northern Triangle, as well as Colombia and Mexico, shows how needs that can be categorized as humanitarian fall through the cracks of the region’s localized crisis management. While the Cold War experience made human rights an alternative to balance *mano dura* or other military approaches, it is not clear whether this is enough to reduce the impact of violence and forced displacement. The need of attention has been followed by the engagement of key humanitarian actors, including the UN High Commissioner for Refugees (UNHCR), the European Union, the World Health Organization (WHO)/Pan American Health Organization (PAHO), and Médecins Sans Frontières (MSF). The International Committee of the Red Cross (ICRC) has made addressing the humanitarian consequences of the violence in Central America an operational priority. How then can the humanitarian localization help the region to increase the humanitarian concern and action? Have humanitarian actors a mandate to respond in what has consistently been labeled as a crime and narcotics crisis better left to the security forces? (Cue and Núnez 2017). In the regional consultation for the 2016 World Humanitarian Summit, OCHA tried to push for a recognition of violence as part of the humanitarian challenges in Latin America, but the national countries blocked this proposal.

Given those large areas of some countries, like the Northern Triangle, are effectively outside of government control, it is clear that humanitarian assistance, delivered by neutral and impartial actors, proportionate in scale, and appropriate to the needs of the affected population, is urgent and relevant. For example, Honduras has recognized the need for protection and assistance to internally displaced people. The country has requested humanitarian aid with the stated aim of increasing institutional budgets for social protection.

There is no doubt that development aid and action are critical for addressing and improving the crisis’s causes, mainly poverty. Balancing the nexus of development, humanitarian and peace support are thus the crux of the problem. In this sense, several organizations, both local and international, have produced interesting results. One example is the European Union’s Peace laboratories, aiming to provide systematic and need-based humanitarian assistance. Another example is the European Civil Protection and Humanitarian Aid Operations grant to UNHCR in 2014 documented forced displacement in Honduras, leveraging awareness among Honduran authorities to incorporate the guiding principles on the human rights of internally displaced populations into national legislation. There is still much room for improvement in attending to humanitarian needs in the region through traditional means, even while the local remains dominant.

### Conclusion: towards a post-localization humanitarian order

Latin America’s history has resulted in a particular, localized order to humanitarian crisis management that notably diverges from the conventional view of North intervention in the South. This order results from the US umbrella against other countries’ intervention and regional efforts to resist through legal frameworks and capacity building. The result was not an absence of crises, which keep hitting the region relentlessly, but consolidated ownership of their understanding and management, making international aid an afterthought to the challenge of protecting the most vulnerable. This Latin-American humanitarian order is more evident in dealing with disasters, in which the region excels, and to a lesser extent in catering for migration needs. The region has a long experience sending, allowing the transit, and receiving different kinds of migrants, usually giving some room for populations to reach their final destination and integrate, going beyond the merely humanitarian. The Latin-American humanitarian order’s major problem has been addressing the consequences of violence, usually associated with gangs and narcotics, causing widespread effects on the population as well as the pandemic, like the recent case of COVID-19. International audiences identify these effects as a humanitarian crisis, but governments consider them part of their national security. If localization’s final aim is something like the Latin-American system, then turning the attention to how to manage issues in which ownership is problematic should be a top priority. Even though the issue of ownership cannot be addressed at large, we mention that the region made progress in recent years; local actors outlined activities that included building organizational capacity in areas like finance and procurement, as well as activities that were more system-wide: performing joint needs assessments, coordinating early warning systems, producing district-level contingency plans, and more. These initiatives improved accountability between local actors and crisis-affected people/communities rather than focusing solely on donor accountability (Bird 2021).

Latin-American countries can be considered a social laboratory for better understanding the Global South view of humanitarian action versus Western concepts and ideas. Two centuries resisting intervention and more than three decades of experience in managing risk and disasters have shown that Latin America can develop important mutual collaboration frameworks during crises, where different actors coexist at different levels. Mediated by bilateral, regional, and multilateral organizations, including NGOs, different forms of “humanitarian interventions” are recognized in the region, and many states have become humanitarian donors (Gómez 2019). Some states have built institutional capacities to organize and deliver international aid, mainly Mexico, Brazil, Chile, and Colombia. On the other hand, this laboratory should be followed by a better understanding of how humanitarian organizations work in the region and how internationalization can positively affect embattled populations. The available diversity of skills, experience, and approaches to humanitarian response makes unclear what the role of parachuting international actors can be. As has been the case for church-related organizations, working through existing networks seems to be the established way of working, but the necessity of disruption cannot be discarded. Talking about complementarity seems more relevant than localization, as humanitarian actors support local responses and push for improved responses from inside.

The humanitarian field in the region is also affected by the global trend of humanitarian “fatigue”: power dynamics, culture, lack of financial resources, the proliferation of regional mechanisms and proliferation of organizations from both development aid, cooperation, and peace building, all creates compelling reasons for the system to be centralized on local governments. Within the current Latin-American centralized humanitarian structure, the proposition of localization is unlikely to result in any changes. Instead, under the new humanitarian order, traditional humanitarian institutions face significant adaptation challenges. As national capacities keep growing, identifying and supplementing unattended gaps and populations left behind, especially without a physical presence in advance, become difficult. Two main options seem available; on the one hand, attending to these needs may require mutating into a human rights-like logic of action, which emphasizes advocacy and denunciation over direct provision. This way of working is well-established in the region, so it is not clear how open it is for new external actors.

On the other hand, humanitarian actors may also become close implementing partners for national governments, aiming to keep raising the standards of response and advising from inside whenever problems are detected. Partnerships sound more attractive and are already taking place, as local UN agencies all over the region, although mainly in more affluent countries, depend on national contributions and contracts (Gómez 2019). However, the loss of independence and complicity when a negligent attitude persists is serious sources of concern. Even the US and European countries have shown the limits of their humanitarian aspirations in the way they are dealing with migrants trying to reach their borders. Humanitarian principles remain a major reason of concern in a future of stronger states without a single easy answer but instead benefiting from multiple case-by-case approaches.

Finally, Latin-American countries need to sustain progress in reaching local communities with emergency preparedness and resilience programs to guarantee effective participation in humanitarian response. The interactions between actors at different levels provide some soft checks and balances, ensuring protection standards are maintained and coordination agreements upheld. The coexistence of multiple approaches to crisis confers flexibility against contingencies, warranting that hard-earned development gains are not lost.

## Data Availability

Not applicable

## Abbreviations

LAC: Latin America and the Caribbean
MD: Monroe Doctrine
OCHA: UN Office for the Coordination of Humanitarian Affairs
WHS: World Humanitarian Summit
NGOs: Nongovernmental organizations
ODA: Official development assistance
DRM: Disaster risk management
CEPREDENAC: Coordination Centre for Natural Disaster Prevention in Central America
CENAPRED: Mexico’s National Centre for Disaster Prevention
CDEMA: Caribbean Disaster Emergency Management Agency
CONRED: Guatemala’s National Coordination for Disaster Reduction
RIMD: Inter-American Network for Disaster Mitigation
SICA: Central American Integration System
CARICOM: Caribbean community
UNASUR: Union of South American Nations
UN: United Nations
MIAH: International humanitarian assistance mechanism
UNHCR: UN High Commissioner for Refugees
WHO: World Health Organization
PAHO: Pan American Health Organization
ICRC: International Committee of the Red Cross

## Footnotes

1 Our argument about the particular characteristics of “local humanitarian” institutions in Latin America is not fully applicable to the rest of the Caribbean, mainly because of the different timing and dynamics of independence from colonial rule. Still, we observe some convergence towards a similar “way of working,” so we include them on “The localized ‘humanitarian’ system in Latin America.” This decision reflects our suggestion that the humanitarian system may be evolving elsewhere towards something like the Latin-American model.

2 Colombia hosted for several years during the two-thousands the world largest internally displaced persons population, attracting international humanitarian organizations, even by invitation from the government. However, the government methodically contained any international pressure or bad press. For instance, according to OCHA’s financial track system, during this period, only one humanitarian appeal was allowed. See Borda Guzmán (Borda Guzmán 2011; Borda Guzmán 2014).

3 O’Sullivan (O’Sullivan 2021) shows how in the 1980s Central American actors had a solid human rights approach, which helped in resisting humanitarian framings.

4
https://periododesesiones.cepal.org/38/sites/default/files/political_declaration_latin_america_and_caribbean-_final_23.10_ing.pdf

5 This section builds on Gómez (Gómez 2021).

6 European colonies, mainly in the Caribbean and North and South America, remain until today.

7 Just as it was attempted in 2019 in Venezuela following the outbreak of the migration emergency

8 Interestingly, from a US diplomatic history perspective, Sanbrailo (Sanbrailo 2016) sees this episode more positively, as the origin of US humanitarian assistance (and food aid). Thanks to an anonymous reviewer for the observation.

9 Other examples of resisting colonialism through the creation of Red Cross societies include Thailand, China, Japan, and Korea (Cook et al. forthcoming).

10 Please note that we are not saying that the region did this in isolation. Indeed, Latin-American scholars were already well connected with academics from around the world and participated in global conversations that served cross-fertilizing both local and global progress on humanitarian crises management, just as we observed about international law and human rights in the previous section, as well as for medicine and public health (Cueto and Palmer 2015), among many other relevant disciplines. Particularly in relation to “La RED,” Allan Lavell, Andrew Maskrey, and Anthony Oliver-Smith are some important names of scholars and practitioners connecting Latin-American and Anglo-Saxon experiences.

11 While different parts of the model described are present in other countries and regions, we argue that the historical process resulting in a different language, national institutions, and nongovernmental actors is different, given the early emancipation from the colonial system, discussed in the previous section. On the other hand, we believe that Latin America suggests the direction towards which the evolution of the humanitarian sector will go, so it is expected to find similar developments elsewhere in the periphery. See for instance Gómez (Gómez 2021).

12 MIAH are regional mechanism built ad hoc for government consultations in disaster risk preparedness and humanitarian responses. They began to operate in 2008 in Mexico and every 2 years. Member states in the region gather to coordinate efforts and exchange knowledge on coordination and cooperation during humanitarian assistance crisis. They are independent from the global platform but pursue at regional level the same goals.

13 It is worth adding that several countries of the region have taken part in INSARAG, the global search and rescue team network, from its origins.
